# Mid-term efficacy and postoperative wound management of laser hemorrhoidoplasty (LHP) vs conventional excisional hemorrhoidectomy in grade III hemorrhoidal disease: the twisting trend

**DOI:** 10.1007/s00423-023-02879-4

**Published:** 2023-04-05

**Authors:** Claudio Gambardella, Luigi Brusciano, Antonio Brillantino, Simona Parisi, Francesco Saverio Lucido, Gianmattia del Genio, Salvatore Tolone, Alfredo Allaria, Salomone Di Saverio, Francesco Pizza, Alessandro Sturiale, Ludovico Docimo

**Affiliations:** 1 Division of General, Oncological, Mini-invasive and Obesity Surgery, University of Study of Campania “Luigi Vanvitelli”, via Luigi Pansini n° 5, 80131 Naples, Italy; 2 Department of Emergency Surgery, “A. Cardarelli” Hospital, Via A. Cardarelli 9, Naples, Italy; 3 Cambridge Colorectal Unit, Cambridge University Hospitals NHS Foundation Trust, Addenbrooke’s Hospital, Cambridge Biomedical Campus, Hills Road, Cambridge, CB2 OQQ UK; 4 Division of General and Emergency Surgery, Asl Napoli 2 nord, Frattamaggiore, Naples, Italy; 5 Proctological and Perineal Surgical Unit, Cisanello University Hospital, Via Paradisa 2, Pisa, Italy

**Keywords:** Hemorrhoidal disease, Laser hemorrhoidoplasty, Excisional hemorrhoidectomy, Postoperative pain, Hemorrhoidal recurrence

## Abstract

**Purpose:**

Hemorrhoidal disease (HD) is a common condition, and several surgical techniques have been proposed to date without being able to achieve definitive consensus on their use and indications. Laser hemorrhoidoplasty (LHP) is a minimally invasive procedure for HD treatment determining the shrinkage of the hemorrhoidal piles by diode laser limiting the postoperative discomfort and pain. The aim of the current study was to evaluate the postoperative outcomes of HD patients undergoing LHP *vs* conventional Milligan-Morgan hemorrhoidectomy (MM).

**Method:**

Postoperative pain, wound care management, symptoms’ resolution, patients’ quality of life, and length of return to daily activity of grade III symptomatic HD patients undergoing LHP *vs* MM were retrospectively evaluated. The patients were followed-up for recurrence of prolapsed hemorrhoid or symptoms.

**Result:**

From January 2018 to December 2019, 93 patients received conventional Milligan Morgan as control group and 81 patients received laser hemorrhoidoplasty treatment using a 1470-nm diode laser. No significant intraoperative complications occurred in both groups. Laser hemorrhoidoplasty patients experienced lower postoperative pain score (*p* < 0.0001) and smoother wound management. After 25 ± 8 months follow-up, the recurrence of symptoms occurred in 8.1% after Milligan-Morgan and 21.6% after laser hemorrhoidoplasty (*p* < 0.05) with a similar Rorvik score (7.8 ± 2.6 in LHP group vs 7.6 ± 1.9 in MM group, *p* = 0.12).

**Conclusion:**

LHP demonstrated high efficacy in selected HD patients guaranteeing lower postoperative pain, easier wound care, higher rate of symptoms resolution, and greater patient appreciation compared to MM, even though it had a higher recurrence rate. Larger comparative studies are needed to address this issue.

## Introduction

Hemorrhoidal disease (HD) is a common anorectal condition severely impacting patients’ quality of life [1, 2]. In case of HD, patients complain of bleeding, pain, and hitching and its treatment ranges from conventional resectional approaches (Milligan-Morgan or Ferguson hemorrhoidectomy) to suspensive ones (Longo procedures and its modifications) [3–5]. All these techniques present proven efficacy in HD, but could be associated to not neglectable complications, such as pain, seromucous discharge, and anal stenosis in Milligan-Morgan hemorrhoidectomy (MM) or defecatory urgency, unbearable pain, tenesmus after hemorrhoidopexy [4–6]. Therefore, because of the fear of postoperative pain and complications and for the need of long postoperative wound healing, mildly symptomatic patients often hesitate and delay undergoing to surgical treatment for this benign disease.

In the last years, several minimally invasive non-excisional procedures including transanal hemorrhoidal dearterialization (THD) and hemorrhoidal artery ligation (HAL) have been proposed to overcome these issues [7]. Laser hemorrhoidoplasty (LHP) is a recent minimal invasive procedure for day-surgery treatment of symptomatic hemorrhoids determining the shrinkage of the hemorrhoidal piles and the tissue degeneration at different depths adopting a diode laser [8–10].

Aiming to define the role of the abovementioned minimal invasive procedure, a retrospective analysis comparing the safety and effectiveness of LHP with conventional MM in treatment of patients with III degrees HD was carried out. The study presented as primary outcome the analysis of postoperative pain, bleeding, wound management, and symptom relief in the first postoperative month. The secondary outcome was the evaluation of medium term recurrence and complications after the procedures within 24-month follow-up and the patients’ HRQoL.

## Methods

### Study design

This study is reported according to the STROBE statement for cohort studies [11]. A retrospective multicentric was conducted to compare the MM hemorrhoidectomy with the minimal invasive LHP, in patients affected by grade III symptomatic HD. It was conducted according to the ethical principles stated in the Declaration of Helsinki. Written informed consent was obtained from all subjects.

### Study setting and study population

From January 2018 to December 2019, all the patients affected by HD and referring to the Division of General Surgery of a Teaching Hospital were considered for the enrollment in the study. Inclusion criteria were age ≥16 years, symptomatic HD of III degree, according to the Goligher classification [12], American Society of Anesthesiologists (ASA) physical status of grade I or II [13]. Exclusion criteria were acutely thrombosed hemorrhoids, patients affected by inflammatory bowel diseases (IBD) involving rectum or anus, recurrent HD, need of manual reduction of an eventual hemorrhoidal prolapse (grade IV), and presence of anal fissure.

All subjects were preoperatively assessed during a specialized coloproctology evaluation [14–17]. Information on bowel function, pregnancies, episiotomy, previous surgery, and associated diseases was recorded. The severity of hemorrhoidal symptoms and the patients’ health-related quality of life was scored using the Rorvik score, 2 weeks before surgery [18]. All patients were addressed to surgery after the failure of the medical treatment consisting in the adjustment of the fecal consistency by balanced assumption of water, fibers, and probiotics to achieve soft stool and the use of bioflavonoids and NSAIDs, for at least two cycles of 40 days in 3 months.

Before surgery, all patients over 50 years underwent pancolonscopy to exclude the presence of neoplasms or inflammatory bowel disease. The other patients received a rectoscopy. All the surgeries were performed by experienced proctologists (over 500 proctological procedures).

Clinical data were collected in a prospective maintain electronic database. Patients who received the conventional Milligan-Morgan hemorrhoidectomy were considered in MM group and patients who received laser hemorrhoidoplasty in LHP group. Only patients actively followed up for at least 2 years, being invited to our outpatient clinic by telephone or mail, were considered. The postoperative follow-up comprised three (approximately at 6–12–24 months) or more appointments for clinical evaluations.

### Surgical technique

#### Milligan-Morgan hemorrhoidectomy

Patients were placed in lithotomy position, and spinal anesthesia was performed. Antibiotic prophylaxis with ceftriaxone (2 g i.v.) was administered. The hemorrhoidectomy was performed by radiofrequency (LigaSure TM Small Jaw® Covidien, Colorado, USA) or ultrasound (Focus Ultracision-Harmonic Scalpel, Ethicon Endo-Surgery® Ohio, USA). The anodermal wedge was incised, eventually removing external fibrosis and/or skin tags when present. Upward dissection started at this level with en bloc excision of mucosal and submucosal layers from the underlying internal anal sphincter up to the anorectal ring. A compressive hemostatic sponge was left in place for 12–24 h. A 24-h elastomeric continuous intravenous analgesic infusion (ketoralac 30 mg/1 ml, tramadol 100 mg/2 ml and ranitidine 300 mg) with a standardized therapy on demand (ketoralac 20 mg/1 ml, dose range 8–20 drops) was indicated.

Management after discharge consisted of dietary modification (e.g., stool softeners and fiber supplements with adequate fluids intake) and standard medical therapy. Wounds were treated by warm water wash 2–3 times per day, followed by oxide zinc powder placement. Postoperative pain after postoperative day 1 was controlled with ketorolac as needed.

### Laser hemorrhoidoplasty

In lithotomy position, a bilateral pudendal nerve locoregional block was performed by administration of ropivacaine (10 ml for each side). A deep sedation, obtained by propofol (2.0 mg/kg i.v.) and associated to the use of a laryngeal mask, was performed. Antibiotic prophylaxis with ceftriaxone (2g i.v.) was administered. A skin microincision of 3 mm was made about 1 to 1.5 cm of distance from the anal verge at the base of each hemorrhoidal node. The probe (1.85 mm of diameter) was driven through the incision in the submucosal tissue until reaching the area underneath the distal rectal mucosa. Then, ten to twelve effective pulses (adjusted to respective node dimensions), 8 watts per 3 s each, of approximately 24 Joule using a 1470-nm diode laser generator (LEONARDO® DUAL 45 Biolitec® Jena, Germany) were fired. Half of them were fired in the submucosal tissue, the others in the intra-nodal compartment determining the shrinkage of the hemorrhoidal piles. The anal wounds were left open. At the end of the procedure, an anal sponge was positioned. After 12 h, the anal sponge was removed, and patients were discharged the day after surgical operation, in case of no postoperative complications, presence of a tolerable pain ≤ 5 with VAS score, and tolerance to oral feeding. Management after discharge consisted of dietary modification (e.g., stool softeners and fibers supplements with adequate fluids intake) and standard medical therapy. Postoperative pain after postoperative day 1 was controlled with ketorolac as needed [10].

### Rorvik score

**Fig. 1:**
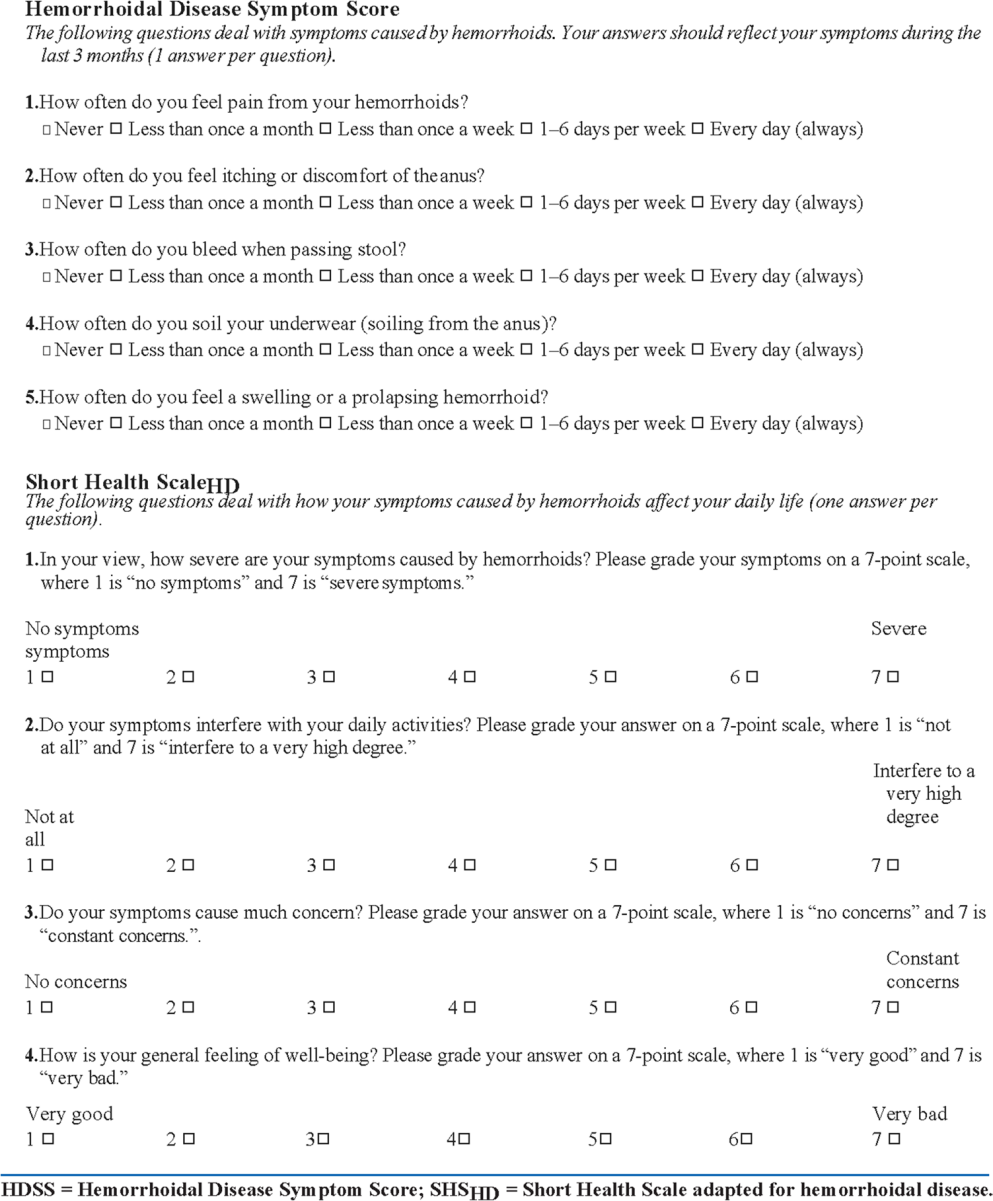
Hemorrhoidal Disease Symptom Score. HDSS, Hemorrhoidal Disease Symptom Score; SHSHD, Short Health Scale adapted for hemorrhoidal disease

To date, no disease-specific measure of health-related quality of life (HRQoL) has been specifically validated for HD. Rorvik et al. proposed a dedicated score composed by Hemorrhoidal Disease Symptom Score (HDSS) and the Short Health Scale (SHS-HD) [16]. The HDSS is based on five different parameters of HD, with a grading from 0 (no symptom) to 4 (daily presence of the symptoms) for each item. The total score of all five parameters was used to evaluate the patient’s condition: 0 indicated the total absence of a symptom, while a score of 20 represented the worst clinical scenario. The Short Health Scale (SHS) is a QoL-based score including information on symptom severity, impact on daily activities, patients’ concerns, and personal feeling of well-being. It ranges from 1 (optimal clinical conditions) to 28 (worst clinical conditions) (Fig. 1) [18–20].

### Wound care management

Local wound care was assessed by recording the modality and weekly frequency of dressing. In details, we recognized two different types of dressing: dressing A, a simple wound cleansing with a shower practiced by the patients himself; dressing B, a wound cleansing with a shower followed by disinfection with Povidone-iodine solution at 10% and sterile gauze to obtain a soft debridement, and use of ointments (i.e., cicatrizants, antibacterial, anti-inflammatory). The dressing B change is more challenging and, at least in the first postoperative days, necessitate the help of a specialized nurse and after of a family member.

This was recorded by means of diaries the patients were asked to complete. The wound care was monitored during the hospitalization and the outpatient follow-up visits until the wound was closed. Wound infection was defined as the presence of local symptoms of suppuration with or without an isolated pathogenic microorganism. Wound healing was defined by complete re-epithelialization recorded by a surgeon who attended the outpatients’ visits.

### Outcome measures

Mean operative time was evaluated in minutes. Postoperative pain, evaluated with the visual analog scale (VAS), and the use of analgesics were evaluated at 6 and 12 h and 1, 3, 7, 14, and 30 postoperative days. Eventual bleeding was evaluated at day 1, 3, 7, 14, 30; it was classified as follows: spontaneous, post-defecatory or no evidence of bleeding. Seromucous discharge, wound infection, and fecal and gas incontinence, assessed using the Cleveland clinic incontinence score for fecal incontinence, were evaluated at 30 days [21]. Time needed to come back to daily activity was also evaluated and expressed in days.

Presence of recurrence and of any postoperative complications (i.e., anal stenosis and fecal incontinence) was assessed at the outpatient visits. Patients were considered to have recurrent hemorrhoidal symptoms when any of the following were recorded: bleeding, itching, pain or discomfort affecting patient’s perception of quality of life, which could either be associated or not to prolapse recurrence.

Anal stenosis was classified according to its severity as mild (tight anal canal assessable by a well-lubricated index finger), moderate (requiring forceful dilatation to insert index finger), and severe (if a pinkie could not be inserted unless a forceful pressure) [22].

Postoperatively, severity of hemorrhoidal symptoms and health-related quality of life (HRQoL) were evaluated via Rorvik score at 30 days and at the postoperative outpatient visits.

At 24 months, patients were asked about the possibility to repeat the procedure in case of recurrence of the disease.

### Study outcomes

The primary outcome of the current study was to analyze the postoperative pain (according to VAS score), the postoperative bleeding, the postoperative wound management, and the symptoms relief in the first postoperative month with LHP in patients with III degrees hemorrhoids compared to MM.

The secondary outcome was the evaluation of medium term recurrence and complications after the procedures within 24-month follow-up and the patients’ HRQoL with LHP and MM.

### Statistical analysis

Statistical analysis was performed via Excel 2011® (Microsoft, Redmont, WA) and through the GraphPad Prism® 9 program (San Diego, California, USA). Categorical data were reported as raw numbers with percentages in parenthesis. Continuous data were reported as means ± standard deviation or as medians with range in parenthesis, according to the distribution. The differences between results were analyzed by the unpaired *t* test if they were summarized as means, the Mann Whitney *U* test if it were summarized as medians, or the Fisher’s exact test if they were reported as percentages.

A probability value of less than 0.05 was considered significant.

## Results

### Study population

**Fig. 2:**
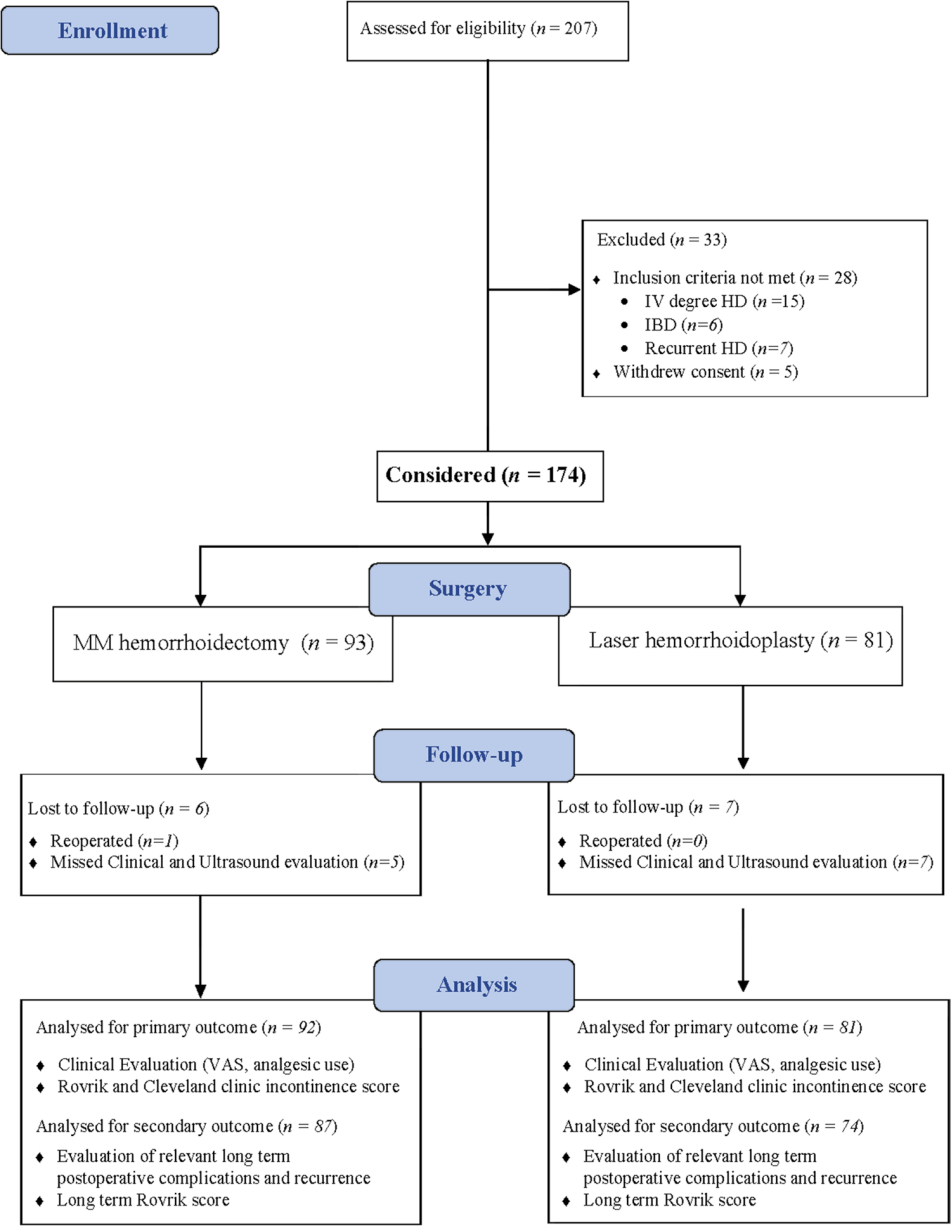
Ninety-three patients received conventional MM (MM group), and 81 patients received minimal invasive LHP procedure (LHP group)

From January 2018 to December 2019 of 207 patients referred for HD, 174 met the inclusion criteria and received HD surgical treatment. Ninety-three patients received conventional MM (MM group), and 81 patients received minimal invasive LHP procedure (LHP group) (Fig. 2). Demographic and pathological findings are detailed in Table 1.

**Table 1:** Patient baseline demographics and characteristic in LHP and MM group

	LHP group (*n* = 81)	MM group (*n* = 93)	*p*
Age (years)	42 ± 10.6	43 ± 12.4	0.066*
Gender (female/male)	51(63%)/30(37%)	53(56.9%)/36(43.1%)	0.648**
Preoperative symptoms			
• Bleeding	81 (100%)	93 (100%)	1.000**
• Hitching	53 (65.4%)	57 (61.2%)	0.572**
• Pain	29 (38.7%)	31 (33.3%)	0.732**
• Prolapsed hemorrhoids	42 (51.8%)	47 (50.5%)	0.862**
Preoperative Rorvik score*	29.8 ± 6.8	30.1 ± 7.2	0.057*

Values are expressed as number of cases with percentages *n* parenthesis or means ± standard deviation

*Unpaired *t* test

**Fisher’s exact test

Mean operative time is detailed in Table 2. The median hospitalization was 1 day in LHP group (1–3 days) and 2 days (2–5 days) in MM group (*p* < 0.0001; Mann Whitney *U* test). No significant intraoperative complications occurred. One patient in MM group was re-operated during the hospitalization (post-operative day 2) for spontaneous bleeding. Seven patients in LHP group and 5 patients in MM group did not attend the outpatients’ visits. Therefore, 174 were considered for the first outcome analysis (81 in LHP group and 93 in MM group), and 162 patients were finally considered in the study for the second outcome analysis (74 in LHP group and 88 in MM group).

**Table 2:** Intra- and peri-operative surgical outcomes after LHP and MM

	LHP group (*n* = 81)	MM group (*n* = 93)	*p*
Mean operative time (minutes)	13 ± 3	18 ± 7	< 0.05*
Number of columns			
• II	15 (18.5%)	16 (17.2%)	0.821***
• III	66 (81.5%)	77 (82.8%)	0.821***
Mean postoperative pain (Visual Analogue Scale)			
• 6 h	3.1 ± 0.8	6.3 ± 1.3	< 0.001*
• 12 h	2.2 ± 0.9	7.4 ± 1.5	< 0.001*
• 24 h	2.1 ± 0.6	7.6 ± 1.3	< 0.001*
• Day 3	1.4 ± 0.4	6.8 ± 1.1	< 0.001*
• Day 7	1.4 ± 0.6	5.4 ± 1.8	< 0.001*
• Day 14	0.8 ± 0.3	3.8 ± 1.5	< 0.001*
• Day 30	0.4 ± 0.2	2.3 ± 1.4	< 0.05*
• Day 45	0.2 ± 0.1	1.8 ± 1.3	< 0.05*
Analgesic use			
• 6 h	21 (19.1%)	78 (70.9%)	< 0.001***
• 12 h	15 (13.6%)	101 (91.8%)	< 0.001***
• 24 h	10 (9.1%)	105 (95.5%)	< 0.001***
• Day 3	2 (1.8%)	98 (89.1%)	< 0.001***
• Day 7	0 (0%)	78 (70.9%)	< 0.001***
• Day 14	0 (0%)	36 (32.7%)	< 0.001***
• Day 30	0 (0%)	5 (6.2%)	< 0.05***
• Day 45	0 (0%)	2 (2.1%)	0.184***
Emergency reintervention	0 (0%)	1 (1.1%)	0.351***
Median time to return to daily activity (days) °	2.1 (range 1-3)	5.8 (range 4–11)	< 0.001**
CCIS*	0	9.7 ± 1.3	< 0.001*
Seromucous discharge	0 (0%)	70 (76%)	< 0.001***
Wound infection	0 (0%)	0 (0%)	NS
Rorvik score	5.1 ± 1.9	11.3 ± 2.7	< 0.001*

Values are expressed as number of cases, means ± standard deviation or medians and range

*CCIS* Cleveland clinic incontinence score

*Unpaired *t* test

**Mann Whitney *U* test

***Fisher’s exact test

### Primary outcome

Mean postoperative pain score evaluated through the visual analog scale (VAS) was significantly lower in LHP group if compared with MM group (*p* < 0.0001; unpaired *t* test) at each follow-up point (Table 2). The percentages of patients who used analgesics after discharge were significantly lower in LHP group (*p* < 0.0001; Fisher’s exact test) (Table 2). One (1.07%) patient in MM group suffered spontaneous bleeding the second day after surgery requiring a surgical revision and 2 blood transfusions. A post defecatory bleeding occurred in LHP group in 34 (41.9%) patients the first day after surgery and in 24 (25.8%) patients on postoperative day 3, resulting in a statistically significative difference (*p* = 0.03; Fisher’s exact test). From the 7th postoperative day, no bleeding event occurred in LHP cohort. In MM group, a post defecatory bleeding occurred in 58 patients (62.3%) the first postoperative day (*p* < 0.0001; Fisher’s exact test), in 32 (34.4%) the second postoperative day (*p* < 0.0001; Fisher’s exact test), and in 39 (41.9%) patients within the first week (*p* < 0.0001; Fisher’s exact test). All the abovementioned cases were conservatively managed. All the other postoperative outcomes (i.e., seromucous discharge, the Cleveland Clinic score, the time to return to daily activities, the local infection, and the Rorvik score) are detailed in Table 2.

Dressing B weekly change median frequency was higher in the MM group during all the observation period, whereas dressing A was necessary in LHP group within the first postoperative week (Table 3).

**Table 3:** Median of dressing changes and modality in LHP and MM groups

	LHP group	MM group	*p*
Dressing A 0–7 days	9 (7–11)	0	< 0.001*
Dressing B 0–7 days	0	21 (18–24)	< 0.001*
Dressing A 8–15 days	0	0	NS
Dressing B 8–15 days	0	18 (16–22)	< 0.001*
Dressing A 16–30 days	0	13 (11–16)	< 0.001*
Dressing B 16–30 days	0	24 (20–28)	< 0.001*
Dressing A 31–45 days	0	15 (14–18)	< 0.001*
Dressing B 31–45 days	0	8 (6–12)	< 0.001*

Data are given as medians with range in parenthesis. Dressing A, a simple wound cleansing with a shower; dressing B, a wound cleansing with a shower followed by disinfection with povidone-iodine solution at 10% and sterile gauze to obtain a soft debridement, and use of ointments (i.e., cicatrizants, antibacterial, anti-inflammatory)

*Mann Whitney *U* test

### Secondary outcome

The overall mean follow-up length was 25 ± 8 months. Hemorrhoidal symptom recurrence was reported in 1 (1.3%), 7 (9.4%), and 16 (21.6%) patients in LHP group at 6 ± 2 months, 12 ± 3 months, and 25 ± 8 months follow-up, respectively. Conversely, hemorrhoidal symptom recurrence was reported in 0 (0%), 3 (3.4%), and 7 (7.9%) patients in MM group at 6 ± 2 months, 12 ± 4 months, and 25 ± 8 months follow-up, respectively. Therefore, at 25 ± 8 months, the percentage of patients with hemorrhoidal symptoms recurrence was significantly higher in LHP group if compared with MM group (*p* = 0.02; Fisher’s exact test) (Table 4).

**Table 4:** Medium-long term surgical outcomes after LHP and MM

*25 ± 8 months follow-up*	LHP group (*n* = 74)	MM group (*n* = 87)	*p*
Anal stenosis	0 (0%)	1 (1.1%)	0.351*
Hemorrhoidal symptoms recurrence	16 (21.6%)	7 (8.1%)	< 0.05*
Hemorrhoidal prolapse recurrence	14 (18.9%)	1 (1.1%)	< 0.001*
Rorvik’s core	7.8 ± 2.6	7.1 ± 1.8	0.564**

Values are expressed as number of cases with percentages in parenthesis or as means ± standard deviation

*Fisher’s exact test

**unpaired *t* test

Hemorrhoidal prolapse recurrence was reported in 2 (2.7%), 6 (8.1%), and 14 (18.9%) patients in LHP group at 6 ± 2 months, 12 ± 3 months, and 25 ± 8 months follow-up, respectively. Hemorrhoidal symptom recurrence was reported in 0 (0%), 0 (0%), and 1 (1.1%) patient in MM group at 6 ± 2 months, 12 ± 4 months, and 25 ± 8 months follow-up, respectively. Therefore, at 25 ± 8 months, the percentage of patients with hemorrhoidal prolapse recurrence was significantly higher in LHP group if compared with MM group (*p* < 0.0001; Fisher’s exact test) (Table 4).

The Rorvik score was 3.8 ± 1.9, 4.7 ± 1.7, and 7.8 ± 2.6 in LHP group at 6 ± 2 months, 12 ± 3 months, and 25 ± 8 months follow-up, respectively. In MM group, it resulted 7.1 ± 1.8, 6.8 ± 2.1, and 7.6 ± 1.9 at 6 ± 2 months, 12 ± 4 months, and 25 ± 8 months follow-up, respectively. No significative difference was found in the mean Rorvik’s score between LHP and MM groups (*p* = 0.12; unpaired *t* test) at 25 ± 8 months follow-up (Table 4).

No patient experienced anal stenosis in LHP group during all the follow-up period. Two (2.2%) patients in MM group reported mild anal stenosis at 6 and 13 months, conservatively managed. One of the latter patients solved the stenosis at 23 months (Table 4).

At 25 ± 8 months, 71 out of 74 (95.9%) patients in LHP group answered positively to the hypothetic possibility of repeating the procedure in case of persistence or recurrence of the disease. Conversely, at 25 ± 8 months, the positive answer was reported by 52 out of 88 (59%) in MM group (*p* < 0.0001; Fisher’s exact test).

## Discussion

To date, the largest study comparing the efficacy of LHP treatment with 980-nm diode laser versus the conventional Milligan-Morgan resection is the one by Naderan et al. [10]. The authors reported, in a cohort of 60 patients, similar results in the effectiveness of the two techniques, underlining the better results of LHP group in terms of postoperative pain and postoperative complications [10].

To the best of our knowledge, the current study is the largest study with the longest follow-up period (25 ± 8 months), comparing the effectiveness and the postoperative complications of HD patients undergoing MM hemorrhoidectomy and LHP. The MM procedure presented longer mean operative time (18 ± 7 min vs 13 ± 3 min, *p* < 0.0001; unpaired *t* test), longer hospitalization (2.2 vs 1.3 days, *p* < 0.0001; Mann Whitney *U* test), and one case of emergency reoperation for spontaneous bleeding. Regarding the first outcome, LHP patients experienced statistically significant lower postoperative pain during all the 30 postoperative days (*p* < 0.0001; unpaired *t* test), use of analgesics (*p* < 0.0001; Fisher’s exact test), and time to return to daily activity (2.1 vs 5.8 days, *p* < 0.0001; Mann Whitney *U* test). Moreover, no patients in LHP group experienced seromucous discharge for the absence of open surgical wounds or fecal incontinence (mean Cleveland clinic incontinence score was 0) in the follow up period. Conversely, seromucous discharge occurred in 70 patients (76%) with a mean Cleveland clinic incontinence score of 9.7 ± 1.3, in MM group. Regarding the wound care management, median weekly dressing B change frequency was markedly higher in the MM group during all the observation period, while LHP group necessitated of only dressing A within the first week. This peculiar dressing care course contributed massively to the minimal invasiveness of the LHP procedure, considering that no patients in the LHP group required an advanced wound-care support. Conversely, for most of the peri-operative period, MM group patients necessitated of the help of a person (i.e., a specialized nurse or a member of the family) to perform the dressing B change. These findings may support the potential impact that laser procedure can have on postoperative management by reducing subjective patient discomfort and postoperative care costs, with a potentially faster return to normal activity. This is also demonstrated by the significantly lower Rorvik score (5.1 ± 1.9 vs 11.3 ± 2.7, *p* < 0.0001; unpaired *t* test), prompted by the minimal invasiveness of the LHP procedure and to the absence of anal wound or excision of tissue below the dentate line, where pain fibers are present [23].

The medium-long term results (secondary outcome), on the other hand, showed a higher recurrence rate after LHP procedure of HD symptoms (21.6% vs 8.1%, *p* < 0.05; Fisher’s exact test) and hemorrhoidal prolapse (18.9% vs 1.1%, *p* < 0.0001; Fisher’s exact test) at 25 ± 8 months. This data was confirmed also by the similar Rorvik score at the latter follow-up period (7.8 ± 2.6 vs 7.6 ± 1.9, *p* = 0.12; unpaired *t* test), highlighting, in fact, a twisting trend: in the first postoperative months, all parameters of the primary outcome (i.e., pain, hospitalization, postoperative bleeding, seromucous discharge, Rorvik score, dressing modality) in favor of LHP procedure, while in the last period analyzed, we observed better results in MM patients in terms of recurrence and symptoms. This is not surprising considering the high short-term efficacy of LHP in symptom resolution associated with very low postoperative pain for the absence of anatomical or functional anorectal impairment, and the supposed higher long-term recurrence rate compared to conventional procedures [8, 10, 24–26].

Noteworthy, the debate on the best surgical treatment for HD is still ongoing within the scientific literature. In fact, while the initial treatment of HD appears consolidated and consisting of lifestyle modifications, dietary supplementation, and administration of phlebotonics, advanced stages are passible of different procedures [20]. The conventional procedures for higher stage HD (i.e., MM hemorrhoidectomy and hemorrhoidopexy) are burdened by possible complications and sequelae; between them the most feared complained are postoperative discomfort and pain [26]. Therefore, in order to satisfy the high request of painless treatment, a wide spectrum of non-excisional and less invasive techniques including transanal hemorrhoidal dearterialization (THD) and hemorrhoidal artery ligation (HAL) have been proposed [7, 27–30].

The most recent alternative techniques for grade II–III HD include the use of laser energy. Hemorrhoidal laser procedure (HeLP) is a non-excisional laser therapy for the treatment of HD, first described in 2009 by Salfi et al [31]. In this procedure, a Doppler identifies the terminal branches of the superior rectal artery, which are coagulated with pulsed laser energy. Differently, LHP, introduced in 2006 by Weyand et al., adopts the concept of laser coagulation in the treatment of vein varicosity borrowed by the endovenous ablation in vascular surgery [32]. The diode laser (wavelength = 1470nm) penetrates up to 2 mm, determining a submucosal denaturation and a controlled shrinkage of the hemorrhoidal tissue. It is selectively and better adsorbed by the hemoglobin, as compared to Nd:YAG laser, and consequently less harmful to the surrounding tissue, preventing any sphincteral lesions [33]. In addition, fibrotic reconstruction generates new connective tissue, which ensures the full mucosa adherence to the underlying tissue. The largest reported series is from Jahanshahi et al., analyzing the feasibility of LHP, with a lower wavelength generator (980 nm), in 368 patients affected by HD [24]. Brusciano et al. adopted the 1470-nm wavelength generator, remarking the short operative time, low postoperative pain, and effectiveness in the treatment of HD of LHP [8]. Moreover, the author highlighted the possibility to perform the procedure with a bilateral pudendal nerve locoregional block associated to a deep sedation assisted by laryngeal mask ventilation, excluding the necessity of spinal anesthesia. Therefore, the procedure can be safely performed even in unfit and elderly patients with several comorbidities. Poskus et al. compared LHP, MM, and sutured mucopexy in a RCT for a total cohort of 121 patients concluding that LHP is a safe, minimally invasive option for hemorrhoids, more effective than sutured mucopexy and less effective than MM [25].

The LHP acts determining a submucosal denaturation and a controlled shrinkage of the hemorrhoidal tissue, but it is possible to argue that over time, a subsequent hemorrhoidal revascularization or neo-angiogenesis could occur. However, considering the mechanism of action of the procedure, the long-term efficacy in case of prolapsed hemorrhoids is limited. Noteworthy, in case of not spontaneously reducing prolapse, in fact, the technique might be not well suited and appears of utmost importance to inform the patients about the possibility of long-term recurrence after LHP, since only scarce data are reported in literature about this issue. Nevertheless, the complete absence of postoperative discomfort and pain probably widely overcomes the latter limitation [8]. In our experience at 25 ± 8 months, the patients were asked about the possibility of repeating the procedure in case of disease persistence or recurrence, and 71 out of 74 (95.9%) patients in LHP group answered affirmatively. Conversely, the affirmative answer was reported by only 52 out of 87 (59.8%) in MM group (*p* < 0.0001; Fisher’s exact test). Probably, in a non-malignant and not life-threatening condition as HD, the most appreciated treatment was not necessarily the most effective and long lasting one, but is most likely based on the combination of effectiveness outcomes, invasiveness of the procedure, and less morbidity it carries with it [23, 27]. Moreover, LHP does not alter the normal anatomy of anal canal and hemorrhoids allowing the possibility of undergoing to a subsequent more invasive surgical treatment in case of recurrence. Finally, it is an easy and reproducible technique, with a short learning curve that allows the surgeon to master the procedure after 3–5 cases [8, 10].

The current study has some limitations to address. First, only patients with HD of III grade were considered in the analysis excluding patients affected by IV where LHP is hardly suitable. Another potential bias is the limited follow-up of 25 ± 8 months, which may not have been long enough for definitive conclusions. The retrospective nature of the study is another limitation, but noteworthy, the scientific literature about the best treatment for HD is still scarce.

Therefore, considering the minimal invasiveness of LHP, its “comfortable” and benign postoperative course, the rapid return to daily activity, the smoother wound care but the unneglectable long-term recurrence rate, probably the key to the success could be to address also mild symptomatic patients in an early stage to this procedure. Further larger comparative studies are needed to better clarify this issue.

## Data Availability

The datasets used and/or analysed during the current study are available from the corresponding author on reasonable request.
